# Evitatious Phenomenon: A Case Illustration and Proposed Research Criteria

**DOI:** 10.7759/cureus.76815

**Published:** 2025-01-02

**Authors:** Jack R Dale

**Affiliations:** 1 Occupational Health, Tasmanian Occupational & Environmental Medicine, Hobart, AUS

**Keywords:** avoidance behaviors, evitatious phenomenon, occupational medicine, occupational psychiatry, return to work

## Abstract

This article introduces the evitatious phenomenon, with a proposed research criteria for behaviors induced by stress-related avoidance in occupational and psychiatric settings. Evitatious Phenomenon is characterized by a conscious desire to avoid stressors, leading to unconscious behaviors that inadvertently result in avoidance. Unlike malingering or factitious disorder, evitatious phenomenon is not marked by intentional deception but rather by an unconscious process, filling a gap in the current understanding of stress-induced behaviors. Three brief illustrative cases are outlined.

## Introduction

The landscape of psychiatric diagnosis often centers around the dichotomy of conscious versus unconscious behaviors and motivations. Psychobehavioral phenomena in clinical practice often present diagnostic challenges. While criteria for malingering, factitious disorder, and functional neurological disorder (FND) are well-established [1-3], they do not account for all possible combinations of conscious and unconscious processes in motivation and behavior. By conceptualizing these processes as a simplistic matrix, it becomes clear that one category remains unexplored: behaviors that are consciously motivated but unconsciously enacted. This paper introduces the evitatious phenomenon to address this gap.

In this conceptual framework, malingering is characterized by both conscious motivation and conscious behavior (CC) [4]. This means that individuals deliberately choose to produce or exaggerate symptoms, fully aware of their actions and the intent behind them, typically to achieve a tangible benefit, such as financial gain or avoidance of responsibility.

In contrast, factitious disorder involves an unconscious motivation paired with conscious behavior (UC) [4]. Here, individuals intentionally create or feign symptoms, but their actions are driven by an unconscious need, such as a desire for attention or to assume the role of a patient, without an external reward.

Finally, FND is associated with both unconscious motivation and unconscious behavior (UU) [2]. In these cases, individuals experience symptoms/behaviors without conscious intent or awareness of the underlying psychological factors contributing to their condition.

This leaves the missing category of conscious motivation and unconscious behavior (CU). The evitatious phenomenon fills this space, describing individuals with a conscious desire to avoid a stressor who engage in behaviors that inadvertently lead to genuine symptoms and avoidance of the stressor, without a conscious recognition of the connection between their actions and the resulting avoidance. Unlike malingering or factitious disorder, the symptoms are not fabricated, and there is no intent to deceive.

While avoidance behaviors have been extensively discussed in the context of anxiety disorders, the evitatious phenomenon represents a unique subset of behaviors. Unlike conventional avoidance strategies where the behavior is intended to evade stressors [5], here, avoidance emerges unintentionally as a byproduct of stress-coping mechanisms. This concept extends Hofman and Hay’s balanced approach to avoidance [5], which acknowledges a spectrum from adaptive to maladaptive behaviors but does not account for behaviors that result in unintended avoidance.

The aim of this case series is to illustrate the evitatious phenomenon through clinical examples and propose research criteria to define its features. It is not a disorder in and of itself but rather a behavior pattern that may come to clinical attention, usually association with other conditions.

Recognizing this phenomenon offers a new perspective for clinicians in addressing stress-induced behaviors and improving patient care, particularly in occupational and psychiatric contexts.

## Case presentation

Case 1: social anxiety disorder and lactose intolerance

A 44-year-old male project manager was referred to a private occupational medicine clinic due to frequent absences from work, particularly on days requiring key presentations to large internal groups or client organizations. During the COVID-19 lockdowns, he had been working from home with no reported problems, but the return to in-person work revealed a new pattern of absenteeism coinciding with high-stakes presentations. There had been no such problems prior to lockdowns.

History

The patient had a history of mild social anxiety disorder estimated to have been present since around age 16, which was diagnosed and managed with around six sessions of cognitive behavioral therapy (CBT) in his early 20s. During that time, he discovered that drinking a warm glass of milk before bed helped him sleep the night before social events. This is something that he eventually stopped doing as he gained confidence and greater control over his symptoms. However, since returning to in-person work, he noted a return of some of his social anxiety symptoms and started taking a warm glass of milk before bed again. However, he found that it was not as effective as when he was younger and tried larger amounts, often consuming between 500 mL and 750 mL of milk on the night before presentations, finding the warm milk soothing. Unbeknownst to the patient, he had mild lactose intolerance, tolerating usually small amounts of lactose in his regular diet but experiencing diarrhea after consuming larger quantities. He rarely consumed milk or milk products otherwise and had not noticed an association previously, likely because in the past the glass of milk was estimated to be less than 250 mL and he rarely consumed other dairy products.

Presentation

The patient reported frequent “gastroenteritis” episodes, characterized by diarrhea (but no nausea or other gastrointestinal symptoms) that typically resolved by midday. These episodes invariably occurred on the morning of significant presentations, leading to his absence from work. Upon detailed history-taking, the link between his milk consumption and the symptoms was identified, and a working diagnosis of mild lactose intolerance was made.

Management and Outcome

The patient was educated about his likely lactose intolerance and the connection between his milk consumption and symptoms. He was advised to avoid milk before bed and was taught relaxation techniques as an alternative strategy to manage pre-presentation anxiety. The patient's general practitioner was informed of the possible lactose intolerance for follow-up and formal diagnosis. A follow-up telephone call with the employer and the patient after two weeks, a month, and three months confirmed that there were no further absences and that the patient had successfully returned to full occupational functioning.

Case 2: anxiety and fainting related to return-to-work after bullying

A 37-year-old woman, employed for a local government organization as a case manager in an open office environment, was referred to a private occupational medicine clinic after experiencing frequent absences from work following a disputed bullying claim. On her first day back at work after the claim was ended, she fainted and was taken to the hospital. Investigations ruled out major cardiovascular, neurological, metabolic, and other causes. Embarrassed by the episode and still apprehensive about returning to work after the bullying claim, she developed significant anxiety about returning to work.

History

The patient reported avoiding breakfast and sometimes dinner the night before work due to anxiety. This pattern of reduced intake led to frequent episodes of pre-syncope, which she described as feeling light-headed (particularly upon standing), a sensation of weakness or fatigue, and some nausea. This caused her to call in "sick" three to four days a week as she believed that she was going to faint again at work. Her anxiety was centered on re-engaging with colleagues and performing her duties after the bullying incident and the fainting episode at work. She felt embarrassed by the fainting episode and did not want it to happen again.

Presentation

Her symptoms were attributed to a combination of reduced oral intake and heightened anxiety. Despite normal medical investigations, it was hypothesized that her pre-syncope symptoms persisted due to these factors, exacerbating her avoidance of work.

Management and Outcome

The patient was treated with fluoxetine 20 mg daily to help manage her anxiety and provided psychoeducation on the relationship between nutrition, hydration, and her symptoms. She was advised to start each morning with a breakfast drink (e.g., Sustagen or Up&Go) and a glass of water (taken in small quantities over a period of time) and to eat small snacks throughout the workday. CBT was provided to address her fears about returning to work. There were two sessions in the first week, then weekly sessions for four weeks, and then a review session four weeks later. Within six weeks of starting treatment, the patient reported no further pre-syncope symptoms or work absences. Fluoxetine was ceased after three months, and at six months, she maintained full occupational functioning without recurrence of problems.

Case 3: anxiety and overtraining prior to sports try-outs

A 12-year-old boy was referred to a private sports clinic with recurrent episodes of severe lower limb pain occurring prior to sports try-outs and matches. Despite significant anxiety about attending these events, the boy was eager to participate. The clinic determined that there was no major musculoskeletal disorder, and, because of the pattern of absences, he was referred to a private occupational medical clinic for advice on managing the psychological component of the presentation (because of previous collaborations between the two clinics).

History

The patient reported a pattern of intense physical practice in the days leading up to try-outs or games. This "cramming" of exercise would often lead to delayed onset muscle soreness (DOMS), which left him unable to walk properly and prevented him from participating in the events. Despite this, he continued the pattern, unaware of the link between his behavior and symptoms.

Presentation

The patient described significant lower limb pain and stiffness on the days of important sports events, accompanied by visible difficulty in walking. These symptoms were temporally related to his overtraining in the days prior. The boy's team would have their training on Monday and matches on Saturday. He would avoid any practice at home, usually until Thursday after school, when he would become worried about the upcoming match. He managed this anxiety by practicing drills on his own and with his brother for 90-120 minutes and then would do squats, which he thought would strengthen his kicks for the game. He estimated that he would do 75-100 squats or until his thighs started to hurt. He would try to do the same on Fridays after school. He did not do any other exercise during the week. By Saturday morning, he would usually have DOMS and would have difficulty walking and would call in sick for the match. He would rest all of the weekend and was sedentary on Monday and would attend training without problems. He first presented at the mid-season break, when there had already been 10 match days and he missed seven of those matches due to lower limb pain and one match due to mild respiratory infection. He attended two matches. It was noted that there had been family engagements (one birthday and one other social event) that meant he could not practice his "cramming" on the weeks in question and he did not have DOMS on the morning of the match. A similar pattern was noted prior to try-outs for a higher-level team.

Management and Outcome

The possible connection between overtraining and his symptoms was explained to the patient and his parents. He was advised to moderate his exercise routine in the days prior to try-outs and games, focusing on regular and balanced training rather than last-minute intense practice. No medications were prescribed. Following these adjustments, the patient reported no further physical problems and was able to attend eight out of the remaining 10 matches (two were missed due to respiratory infection and a family event). However, significant pre-match anxiety persisted and he was referred to a child psychologist for further support, which he started after the season was over.

## Discussion

Defining the evitatious phenomenon

The evitatious phenomenon represents a subset of behaviors driven by a conscious desire to avoid stressors but manifested through unconscious coping mechanisms. This phenomenon is characterized by several key features, as discussed in the following.

Conscious Motivation

Conscious motivation refers to a desire to avoid a specific future stressor (e.g., workplace obligations, social events).

Stress-Related Behavior

This refers to engagement in behaviors that are brought on by the stress or worry and may provide a short-term relief from stress or worry but are not directly intended to prevent the stressor (e.g., excessive gaming, over-eating, or intensive exercise).

Symptom Development

Genuine physical or psychological symptoms emerge as a direct consequence of the self-soothing behavior (e.g. fatigue, headaches, DOMS, gastrointestinal symptoms, pre-syncope).

Inadvertent Stressor Avoidance

Symptoms inadvertently lead to the avoidance of the stressor, fulfilling the original motivation unconsciously.

Behavior-Symptom Disconnect

The patient does not consciously recognize the link between their behaviors and the resulting symptoms that facilitate avoidance.

Resolution Upon Awareness

Once the patient becomes aware of the connection between their behaviors and symptoms, the phenomenon typically resolves quickly, often with minimal intervention. Awareness in this context refers to the patient recognizing the causal link between specific behaviors (e.g., dietary habits, overtraining, or avoidance strategies) and the resulting symptoms or functional impairments. Indicators of this awareness may include the patient being able to articulate this connection during clinical discussions, demonstrating insight into the role of their behaviors in symptom development and exhibiting a readiness to modify these behaviors once the connection is explained. This awareness can be assessed qualitatively through history-taking and follow-up conversations. It is possible that it could also be assessed quantitatively through validated psychological tools that measure health-related insight and behavioral change readiness; however, this has not yet been attempted in this context. Importantly, achieving this level of awareness is often the turning point for resolving the phenomenon and restoring functional capacity. The patient being able to take steps to address this based on insight is a key differentiating factor between evitatious phenomenon and malingering.

The three cases illustrate the evitatious phenomenon by showcasing behaviors consciously motivated to manage stress or achieve goals but unconsciously enacted in ways that produce genuine symptoms and result in unintentional avoidance. In case 1, a man's attempt to soothe social anxiety by consuming milk led to unrecognized lactose intolerance symptoms, causing him to miss work presentations. Case 2 features a woman who, consciously avoiding food due to anxiety about returning to work, developed pre-syncope symptoms that facilitated avoidance without recognizing the connection. In case 3, a boy’s overtraining before sports try-outs, driven by performance anxiety, resulted in DOMS that prevented participation. In all cases, the individuals were unaware of how their behaviors were linked to their avoidance, fulfilling the criteria of the evitatious phenomenon as distinct from other psychobehavioral conditions.

Differentiation from related conditions

The evitatious phenomenon is proposed as a distinct concept, setting it apart from other conditions involving avoidance behaviors. Malingering involves the intentional fabrication or exaggeration of symptoms for external gain, while factitious disorder is characterized by the deliberate production of symptoms without any apparent external rewards. In contrast, FND involves the unconscious emergence of genuine symptoms that are not directly linked to self-soothing behaviors under conscious control [1-4].

A summary table of these distinctions is provided in Table 1.

**Table 1:** Comparison of evitatious phenomenon, malingering, factitious disorder, and functional neurological disorder

Defining Characteristics	Evitatious Phenomenon	Malingering	Factitious Disorder	Functional Neurological Disorder
Conscious motivation	Yes (avoid stressor)	Yes (gain/avoidance)	No	No
Unconscious behavior	Yes (indirect)	No	No	Yes
Symptom development	Genuine physical or psychological symptoms (e.g., muscle soreness, headache, pre-syncope, gastrointestinal)	Intentionally produced or exaggerated symptoms	Intentionally produced symptoms, but without obvious external incentives	Genuine symptoms, but psychological in origin
Awareness of behavior-symptom connection	No	N/A	N/A	No
Inadvertent avoidance of stressor	Yes	No	Varies	Varies
Exclusion of intentional deception	Yes	No	No	Yes
Functional impact	Yes	Varies	Varies	Yes
Resolution upon awareness	Quick resolution but may need help	N/A	N/A	Usually not

Avoidance is a common coping strategy that can be associated with both positive and negative adaptation [5]. The Evitatious Phenomenon is a specific type of inadvertent avoidance behavior that causes symptoms that allow an opportunistic avoidance of the stressor. It can be differentiated from the avoidance or "impairment" found in malingering, factitious disorder, and FND by the clear stressor-induced behavior that often leads to symptoms that are opportunistically (albeit often legitimately) used to avoid the stressor.

It should also not be confused with self-sabotage behavior. Self-sabotage often involves a degree of intentionality, where individuals consciously or semi-consciously engage in behaviors that disrupt their goals, for example as seen in borderline personality disorder [6]. In contrast, the evitatious phenomenon arises from behaviors that unintentionally lead to avoidance, without conscious recognition of the connection. This nuance is critical for therapeutic intervention, as patients benefit from psychoeducation rather than approaches aimed at addressing intentional self-sabotage.

Clinical implications

Early recognition of the Evitatious Phenomenon can prevent unnecessary medical investigations and perhaps reduce healthcare costs. Tailored psychoeducation and insight-oriented therapy can swiftly resolve these behaviors once the patient recognizes the connection between their actions and symptoms. Care should be taken to identify any underlying psychiatric condition that may be leading to the need for the self-soothing or other behavior.

Proposed research criteria

The following criteria are proposed to facilitate the identification and study of the evitatious phenomenon: (1) conscious desire to avoid a future stressor, (2) engagement in self-soothing or other behaviors not intended to directly prevent the stressor, (3) development of genuine symptoms as a consequence of these behaviors, (4) Symptoms inadvertently lead to avoidance of the stressor, despite the behavior not being intended as a means of avoidance, (5) lack of conscious recognition of the behavior-symptom connection, (6) exclusion of intentional deception, and (7) rapid resolution of the behavior pattern upon awareness (although simple interventions may be required to help).

Future research

By identifying a distinct quadrant in the conscious-unconscious behavior matrix, these criteria may help expand our understanding of stress-induced behaviors. The criteria highlight the role of stress-motivated behaviors that cause inadvertent symptoms and unintended avoidance in causing or perpetuating temporary functional impairment, providing clinicians with a new diagnostic and therapeutic lens.

## Conclusions

The evitatious phenomenon offers a novel perspective on the interplay between conscious and unconscious processes in motivation and behavior, addressing a gap in existing diagnostic frameworks for psychobehavioral conditions. This concept provides a clearer understanding of behaviors that are consciously motivated yet unconsciously enacted, distinguishing them from malingering, factitious disorder, and FND. Through clinical examples, the relevance of recognizing this phenomenon in psychiatric and occupational contexts is illustrated. While these findings are preliminary, they highlight the need for further research to validate and refine the proposed criteria. By broadening the understanding of stress-related behaviors, this framework may contribute to more effective clinical practices.
